# Radiogenomics-Based Risk Prediction of Glioblastoma Multiforme with Clinical Relevance

**DOI:** 10.3390/genes15060718

**Published:** 2024-06-01

**Authors:** Xiaohua Qian, Hua Tan, Xiaona Liu, Weiling Zhao, Michael D. Chan, Pora Kim, Xiaobo Zhou

**Affiliations:** 1 Department of Radiology, Wake Forest School of Medicine, Winston-Salem, NC 27157, USA; 2 Department of Bioinformatics and Systems Medicine, McWilliams School of Biomedical Informatics, The University of Texas Health Science Center at Houston, Houston, TX 77030, USAxiaona.liu@uth.tmc.edu (X.L.); pora.kim@uth.tmc.edu (P.K.); 3 Department of Radiation Oncology, Wake Forest School of Medicine, Winston-Salem, NC 27157, USA

**Keywords:** glioblastoma, 2YS survival rate, PTP, TTP, radiogenomics

## Abstract

Glioblastoma multiforme (GBM)is the most common and aggressive primary brain tumor. Although temozolomide (TMZ)-based radiochemotherapy improves overall GBM patients’ survival, it also increases the frequency of false positive post-treatment magnetic resonance imaging (MRI) assessments for tumor progression. Pseudo-progression (PsP) is a treatment-related reaction with an increased contrast-enhancing lesion size at the tumor site or resection margins miming tumor recurrence on MRI. The accurate and reliable prognostication of GBM progression is urgently needed in the clinical management of GBM patients. Clinical data analysis indicates that the patients with PsP had superior overall and progression-free survival rates. In this study, we aimed to develop a prognostic model to evaluate the tumor progression potential of GBM patients following standard therapies. We applied a dictionary learning scheme to obtain imaging features of GBM patients with PsP or true tumor progression (TTP) from the Wake dataset. Based on these radiographic features, we conducted a radiogenomics analysis to identify the significantly associated genes. These significantly associated genes were used as features to construct a 2YS (2-year survival rate) logistic regression model. GBM patients were classified into low- and high-survival risk groups based on the individual 2YS scores derived from this model. We tested our model using an independent The Cancer Genome Atlas Program (TCGA) dataset and found that 2YS scores were significantly associated with the patient’s overall survival. We used two cohorts of the TCGA data to train and test our model. Our results show that the 2YS scores-based classification results from the training and testing TCGA datasets were significantly associated with the overall survival of patients. We also analyzed the survival prediction ability of other clinical factors (gender, age, KPS (Karnofsky performance status), normal cell ratio) and found that these factors were unrelated or weakly correlated with patients’ survival. Overall, our studies have demonstrated the effectiveness and robustness of the 2YS model in predicting the clinical outcomes of GBM patients after standard therapies.

## 1. Introduction

Glioblastoma multiforme (GBM) is the most common and aggressive primary brain tumor in adults, with a median survival of 14–16 months and an average 2-year survival rate of 26–33% [1]. The current standard of care is surgical resection followed by radiotherapy and adjuvant chemotherapy with temozolomide (TMZ) [2]. Although radiochemotherapy with TMZ is superior to radiotherapy alone in the treatment of GBM, this intensified treatment leads to an increased rate of pseudo-progression (PsP) [3]. PsP is a subacute and post-treatment reaction of imaging changes at the tumor site or resection margins. The contrast enhancement and considerable vasogenic edema on the conventional postoperative monitoring MRI can mimic early tumor progression, making early diagnosis difficult for the GBM patient through the current imaging techniques, although these imaging changes subsequently regress or remain stable [4,5].

Although pathological confirmation is the reference standard in the clinical management of GBM treament, it is not desirable in clinical practice due to the requirement of a second surgery. Follow-up imaging has been employed to make a diagnosis based on the morphological changes of suspected lesions. To achieve a satisfactory diagnostic accuracy, it usually takes several months in the current clinical practice, thus affecting the clinical management of GBM patients. Therefore, the early diagnosis and prediction of PsP are critical for the prognosis of GBM patients. Many efforts have been made to explore imaging signatures or genomics biomarkers for the diagnosis or prediction of the clinical outcome of GBM patients. Perfusion parameters, especially tumor blood volume, have been used as a prognostic signature for disease progression or survival [6,7,8,9]. Other morphologic imaging features (i.e., the non-enhanced and contrast-enhanced tumors, necrosis, and edema) also serve as prognostic markers of GBM [10,11]. Several genomics biomarkers, such as Methylguanine methyltransferase (MGMT) promoter methylation [12], Ki67 expression [13], isocitrate dehydrogenase (NADP(+)) 1 (IDH1) mutation [14], and p53 mutation [15], have been reported as associated with the development of PsP in GBM patients. Among these biomarkers, the MGMT promoter methylation has attracted a lot of attention in the context of this disease [3,12,13,16,17,18]. However, its predictive potential in PsP remains debatable [14,19,20,21,22]. Some machine learning and deep neural network methods that can predict the survival rate of GBM patients based on imaging data and clinical features have been developed [23,24,25,26]. However, there are no systematical and comprehensive approaches for the accurate stratification of PsP and TTP and the prediction of clinical outcomes of GBM patients receiving standard treatment. According to the previous clinical data analyses, the patients with PsP had superior overall and progression-free survival rates [27,28]. However, most of the previous studies did not investigate the association between imaging signatures or genetic biomarkers and patient survival. In our study, by analyzing the private dataset in our institute, we found that there was no significant difference in patients’ survival between PsP and TTP groups diagnosed by clinicians, highlighting the difficulty in obtaining accurate diagnosis using conventional clinical methods. An incorrect diagnosis of a PsP could result in the erroneous termination of an effective treatment, with a potentially negative influence on patients’ survival. Therefore, a novel and reliable diagnostic and predictive risk model with clinical relevance is required for distinguishing these two groups of GBM patients.

Radiogenomics research mainly refers to the relationship between the patient genetics and imaging characteristics [29]. Exploring this relationship can be useful for the accurate assessment of the risk of GBM patients in the development of PsP or TTP. However, comprehensive analysis imaging signatures and genomics biomarkers for constructing a predictive survival risk model are still challenging. For example, Beck et al. found that the prognostic model score from the image-based model was strongly associated with patients’ overall survival, but independent of the clinical, pathological, and molecular factors of breast cancer [30]. Furthermore, gene expression profiles may provide more predictive power of the disease outcome than standard systems based on clinical and histologic criteria [31,32].

In this study, we extracted image features from diffusion tensor imaging (DTI), identified the significant genes, performed the survival analysis of the 2YS model on the Wake dataset and TCGA dataset, and assessed the significance of selected genes. We developed a novel radiogenomics-based 2-year survival (2YS) risk predictive model using a machine learning approach to stratify patients into high- and low-survival risk groups. The radiogenomics study was used to integrate the imaging characteristics and genetic profiles of patients to determine the significant genes and 2YS model for stratifying GBM patients into survival-associated groups. For the radiogenomics study, we applied the dictionary learning method and feature selection scheme to derive the discriminative imaging features from the diffusion tensor imaging (DTI) of PsP and TTP. We explored the relationship between the identified imaging features and differentially expressed genes using the sparse regression model. The genes most relevant to the imaging features were considered as the significant genes. We applied the significant genes as features to build a 2YS predictive model. The patients were divided into high- and low-survival risk groups based on their 2YS scores. Our predictive model was developed based on the dataset from Wake Forest University and independently validated using the public dataset of TCGA. The 2YS as a measure is independent of clinical indicators, such as age, race, and Karnofsky Performance Scale (KPS), but is associated with the IDH1 mutation. Our study contributes an integrative analysis through the combination of imaging characteristics and gene expression data with patients’ survival to discriminate PsP and TTP. Our developed 2YS predictive model will provide a novel tool for personalized diagnosis and care for GBM patients.

## 2. Materials and Method

**Figure 1:**
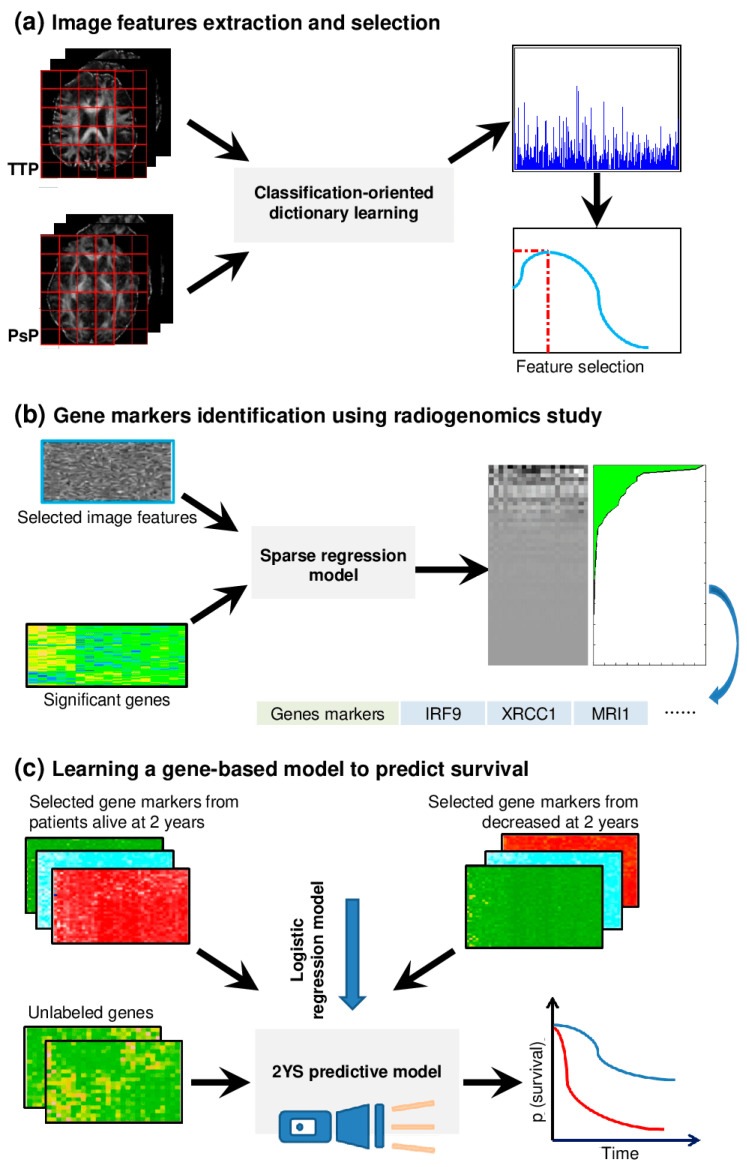
Overview of the radiogenomics study pipeline and prognostic model building procedure. The different colors in the survival curves indicate that the patients are divided into two groups based on the output of the 2YS predictive model. This retrospective study was approved by the Institutional Review Board of Wake Forest School of Medicine. We acquired data from two independent cohorts: the Wake Forest School of Medicine, namely, the Wake dataset, and the Cancer Genome Atlas (TCGA, http://cancergenome.nih.gov/ (accessed on 12 January 2015)).

***The pipeline of the 2YS model.*** Figure 1 shows the overview of the radiogenomics study pipeline and building procedure of the prognostic model. The discriminative imaging features were derived from the DTI of PsP and TTP patients’ groups (Figure 1a). We explored the relationship between the identified imaging features and differentially expressed genes of two treatment phenotypes using the sparse regression model. The genes most relevant to the imaging features were considered the significant genes (Figure 1b). The prognostic model was constructed based on the significant genes from the patients who have survived for 2 years and died during the 2 years after therapy. The 2YS score was applied to stratify GBM patients into high- or low-risk groups.

### 2.1. Data Collection

In the Wake Forest database, more than 200 GBM patients underwent standard multimodal treatment, including the surgical resection and then concurrent radiotherapy and chemotherapy with the alkylating drug TMZ. Along with radiochemotherapy, the patients underwent DTI scans (scanner: SIMCGEMR, GE Medical systems) every three months for post-surgical monitoring. The clinical diagnosis for PsP and TTP was mainly dependent on morphological changes in lesions on the follow-up imaging and clinical knowledge of the physicians. We retrospectively selected DTI scans for the patients diagnosed with PsP or TTP. As a result, we collected both DTI data and electronic clinical records for 84 patients (23 with PsP and 61 with TTP). Genetic data were collected from 42 patients and profiled using the Affymetrix HuEx-1_0-st-v2 array, Illumina HumanMethylation450 BeadChip, and Affymetrix whole genome SNP 6.0 array. The corresponding electronic clinical records, including age, sex, date of surgery, and date of death, were reviewed to determine patient characteristics and treatment outcomes. The Wake dataset was applied to determine the significant genes as features and train the predictive model for survival. The TCGA dataset was applied to validate the survival predictor. Combining gene expression data and survival information for each patient, we collected 158 cases with genetic data and electronic clinical records from TCGA. Image feature extraction was conducted using the dictionary learning approach.

The purpose of deriving imaging features is to conduct the radiogenomics study for the determination of significant genes, thereby emphasizing their relationship with the progression of PsP or TTP. To achieve this goal, we extracted and selected discriminative features from DTI for the classification of these two phenotypes, since DTI showed power in distinguishing these two phenotypes in previous studies [33,34]. However, the segmentation of the suspected lesion area on DTI is a challenging task; thus, we proposed a dictionary learning strategy for capturing the fine difference between the two classes, avoiding the DTI segmentation. The feature extraction and selection procedures comprised three steps: preprocessing DTI data, discriminative dictionary learning, and feature extraction and selection.

### 2.2. Preprocessing DTI Data

We corrected current-induced distortions and subject movements of DTI and conducted skull stripping to obtain the brain images. The fractional anisotropy (FA) value of each voxel in DTI was calculated using FSL software and registered to the standard brain template FMRIB58 in FSL. The identical resolution and number of slices for each registered volumetric FA datum were confirmed.

### 2.3. Discriminative Dictionary Learning

Although the FA values are lower for the lesion sites of PsP than for those with TTP [33], the difference between them is subtle. We previously developed a discriminative dictionary learning scheme [35] to capture the subtle distinctions between PsP and TTP. Specifically, we directly learned specific dictionaries for PsP and TTP to gain classification-oriented characterizations and simultaneously learned shared dictionaries to describe the common features. Intuitively, PsP-specialized dictionaries with the shared dictionaries can outperform TTP-specialized dictionaries with shared dictionaries in the representation of the PsP data; thus, we applied a dual-sparse encoding strategy on each identical case to obtain the discriminative sparse coefficients [35]. Then, we used the sparse coefficient corresponding to the specific dictionaries to construct a new discriminative sparse matrix, which contains more discriminative information attributed to discarding the shared patterns.

### 2.4. Feature Extraction and Selection

Sparse coefficients are the weights of different atoms in the dictionary for the representation of the data. We applied the max-pooling technique to extract the biggest sparse coefficients (i.e., biggest contributions) of each corresponding atom in an indivial patient as imaging features from the constructed discriminative sparse matrix [35].

Since the distinctions between PsP and TTP on DTI are subtle, we applied a discriminating capability of a classifier (DX) score-based feature scoring system to determine the most relevant features [36,37], with a high discrimination power between PsP and TTP. The effectiveness and efficiency of this feature selection method have been confirmed in our previous studies [35,36,37]. Briefly, ${DX}_{score}$ is used to measures the degree of dissimilarity between positive and negative samples for each individual feature, normalized by the sum of variance in the respective sample types. It can be mathematically defined as

(1) $$DX_{score}=\frac{{\left(m_{pos}-m_{neg}\right)}^{2}}{{d_{pos}}^{2}+{d_{neg}}^{2}}$$

where $m_{pos}$ and $m_{neg}$ are the mean values of the positive and negative samples, respectively, while $d_{pos}$ and $d_{neg}$ are the corresponding standard deviations. Then, we constructed a feature set by consecutively adding each feature with ${DX}_{score}$ from high to low and assessed its classification performance by 10-fold cross validation (CV) using the well-established SVM tool LIBSVM [38]. We also conducted a grid search on the radial basis function parameters (i.e., γ and the trade-off coefficient C) for each fold experiment. We identified the best accuracy and its corresponding feature set, which served as the optimal feature set.

### 2.5. Identification of Significant Genes Using Radiogenomics Study

To identify a set of significant genes closely related to the clinical treatment outcomes, we utilized a Wilcoxon rank sum test to compare the gene expression levels in the PsP and TTP groups and selected the significantly differentiated expressed genes with *p*-values less than 0.005. Second, we applied a sparse regression approach to reveal the associations between the gene expression profiles and imaging features extracted by the dictionary learning. We also developed a low-rank sparse regression model to effectively reduce the redundant information, since different imaging features are interrelated to each other and their effects during the association process could be overlapped [39]. According to the associated map (i.e., weight coefficients), we identified a compact set of genes whose expression values were closely related to the imaging features. Specifically, we calculated the overall weights for the expression of each gene with respect to all imaging features. The genes with top-ranked weights were selected as the significant genes. These genes were highly correlated to the imaging features.

### 2.6. Construction of a 2-Year Survival (2YS) Model with Machine Learning

We constructed a predictive model based on gene expression data to predict the 2YS score and then divide the patients into two categories of high- and low-risk, associated with survival time. We utilized the logistic regression model to build this prediction model with the input variables of significant genes’ expression values and the output of the 2YS probability of each patient. Since the complete survival records were available in this study for GBM patients, it was easy to obtain the individual 2YS probability. Consequently, this trained model was used to predict the 2YS score for each patient with gene expression data. To differentiate these GBM patients into different risk subtypes, we used an exhaustive search scheme to identify a threshold for the 2YS score. The patients were classified into subtypes based on the candidate threshold and the differences between subtypes, as assessed by the *p*-value in the Log-rank test. The smallest *p*-value (i.e., the most significant survival difference between the two groups) in all of these subtypes was determined, and the corresponding candidate threshold was chosen as the optimal threshold for this model. In this way, we built the 2YS predictive model and determined the threshold, thereby stratifying the GBM patients into high- and low-survival risk groups. A total of 39 cases from the Wake dataset were used to train this predictive model. We applied this model to the TCGA dataset of 158 cases from multi-institutions for validation. We also conducted random experiments on the TCGA dataset to confirm the performance of this model. Briefly, the 158 cases of the TCGA dataset were randomly split into two folds as the training and validation sets. The Log-rank *p*-value was calculated on the classified high- and low-risk groups. Kaplan–Meier curves were applied to illustrate the survival difference.

### 2.7. Analysis of the Relevance between 2YS Scores and Clinical Factors

We conducted a correlation analysis of 2YS scores and clinical factors for the Wake and TCGA datasets. GBM patients were divided into PsP or TTP based on their 2YS scores, and then the correlation of individuals with each clinical variable was calculated. The trends of continuous variables (such as ages and the tumor/normal cell ratio) were evaluated using the Kolmogorov–Simonov test, and the trends of a discrete variable, including gender, race, and MGMT, were tested by a Fisher exact test. Although most of these clinical factors or measurements were identical in the Wake and TCGA datasets, the consistency of the correlations between the two datasets was not emphasized due to the diversity of the clinical records in different cohorts. To assess the prognostic value of 2YS scores in the context of other clinical factors, we also conducted a multivariate Cox proportional hazards analysis for overall survival on the two cohorts. The statistical significance of each variable in the multivariate Cox proportional model was evaluated by calculating each variable’s Wald statistic and associated *p* value.

### 2.8. Identification of Significant Gene Features in the 2YS Model

To identify the powerful features (i.e., genes) of the 2YS model, we conducted a bootstrap analysis of the TCGA dataset. One thousand bootstrap iterations were performed to generate 95% confidence intervals (CIs) for predicting the coefficient estimates for each gene’s expression value. If 95% CIs for an individual gene are all positive or negative, the corresponding features of this gene are considered as the significant features for the 2YS predictor. Thus, the identified significant features were used to predict clinical treatment progression.

## 3. Results

### 3.1. Extraction of Image Features from DTI

**Figure 2:**
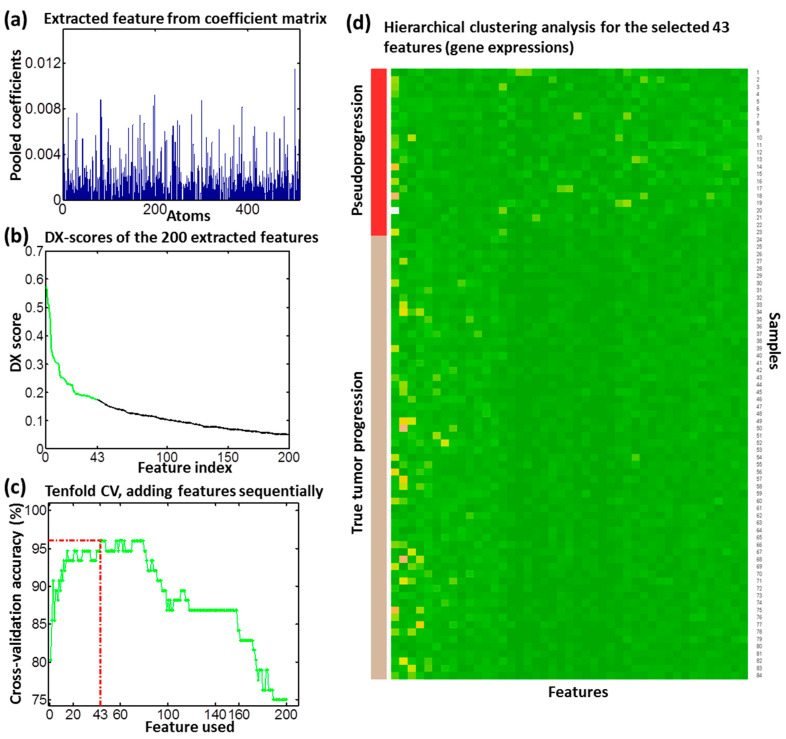
The performance of the extracted imaging features by the dictionary learning of DTI. Panel (**a**) shows the extracted imaging features using the max-pooling technique and (**b**) shows the DX scores of these features, where the green color refers to the top 43 features selected. Panel (**c**) shows a tenfold CV analysis for the features added in one-by-one according to (**b**). The inclusion of the 43 top-ranked features yielded the highest CV accuracy. Panel (**d**) shows the hierarchical clustering analysis of the 43 selected features. Samples with labels from 1 to 23 are PsP cases; those with labels from 24 to 84 are TTP cases.

To improve the computational efficiency, we cropped the original resolution (256 × 256) of images to 164 × 143. We set the following empirical parameters according to our previous studies [35]. The patch size was set as 13, the PsP- and TTP-specialized dictionary sizes were set as 100, the shared dictionary size was set as 10, and the number of non-zero coefficients was set as 10. Using the classification-oriented dictionary learning algorithm, we derived PsP- and TTP-specific dictionaries and shared dictionaries. With the dual sparse encoding scheme, we then excluded coefficients of the shared dictionaries and used the remaining data (i.e., coefficients from the specific dictionaries) to construct a new sparse coefficient matrix. We then applied the max pooling scheme to extract features from the coefficient matrix, as shown in Figure 2a. The pooled features (i.e., a histogram for all atoms) represent the greatest contributions of each corresponding atom in a specific case.

To obtain the most discriminative features, we assessed these pooled features using proposed feature scoring systems [36,37]. The pooled features were sorted by ${DX}_{score}$ (Figure 2b), which indicates the features with higher scores have a better ability in discriminating between PsP and TTP. We sequentially added these ranked features to form a feature set and evaluated their classification performance by 10-fold CV (Figure 2c). We determined the 43 top-ranked features with the best classification accuracy as the optimal feature set, whose discriminative ability was further confirmed by hierarchical clustering analysis (Figure 2d). The sample numbers 1 to 23 and 24 to 84 represent the PsP and TTP cases, respectively. The hierarchical clustering analysis shows a significant difference between the two phenotypes.

### 3.2. Identification of the Significant Genes

**Figure 3:**
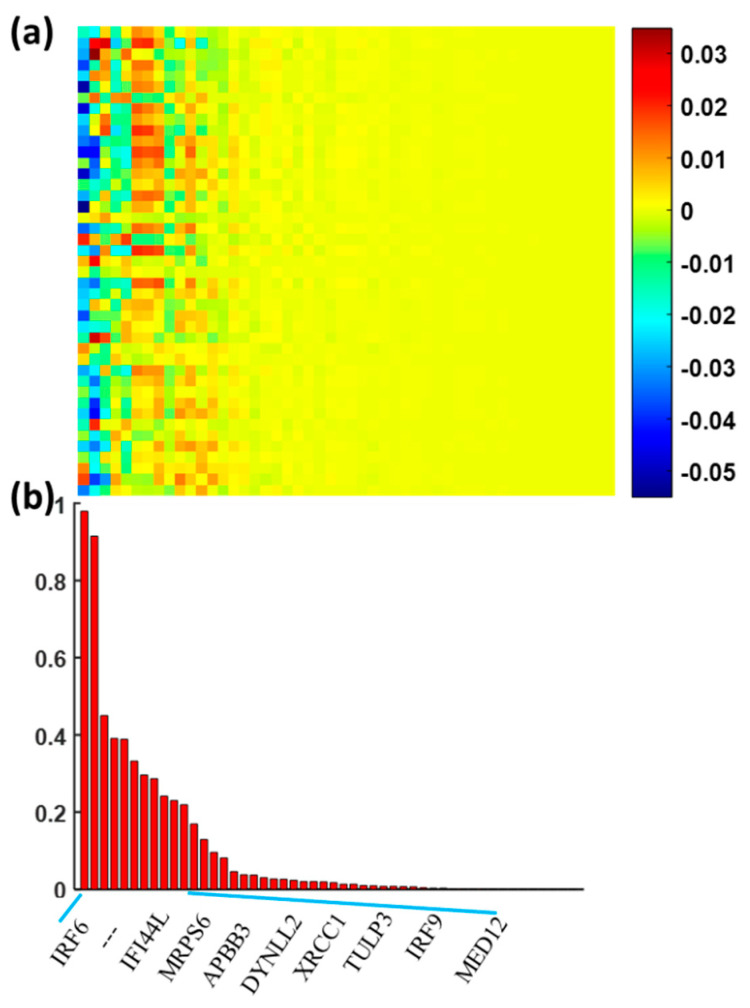
The association of the imaging features with the differentially expressed genes. Panel (**a**) shows the overall weight map of 119 genes relative to 43 imaging features and panel (**b**) shows the genes with top-ranked weights across imaging features.

In our previous radiogenomics study, we identified 119 differentially expressed genes with *p <* 0.005 using the Wilcoxon rank sum test (Supplementary Table S1). We applied the sparse regression model to assess the relationship between the 119 genes and 43 imaging features from DTI. Figure 3a shows the association map between the genes and imaging features. We calculated the overall weights of each gene relative to the 43 imaging features and sorted them from left to right in Figure 3. Twenty-three genes with a top-ranked weight sum were identified as the significant genes (Supplementary Table S2), which were highly correlated with the imaging features. The data from Figure 2c,d demonstrated the discriminative ability of 43 top-ranked features.

To confirm the biological and clinical relevance of our identified significant genes, we conducted a functional analysis of these 23 genes by checking their enriched signaling pathways and disease relevance. These genes were most enriched in the interferon and DNA break repair-related pathways, indicating their extensive participation in the inflammation and cell cycle processes. These results further confirm that the radiogenomics approach is promising in identifying genes with high clinical relevance. More details are provided in the Supplementary Materials.

### 3.3. Survival Analysis of the 2YS Model on the Wake Dataset and TCGA Dataset

**Figure 4:**
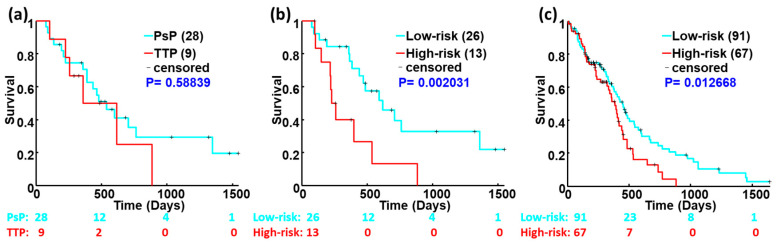
Kaplan–Meier survival analysis based on the clinical diagnosis (**a**) and 2YS scores using the Wake dataset ((**b**), *n* = 39) and TCGA dataset ((**c**), *n* = 158).

We applied the Wake dataset of 39 cases as the training data to build the 2YS prognostic model for the prediction of the binary outcomes. The patients were divided into high- and low-risk groups based on their 2YS scores. The Kaplan–Meier survival curves in Figure 4b show the significant survival difference between the two groups (Log-rank *p* = 0.0020) using the Wake dataset. We tested this model using the TCGA dataset of 158 cases, which was not used in constructing the prognostic model. The survival difference between high- and low-risk groups were statistically significant with a Log-rank *p* = 0.0127, as shown in Figure 4c. Kaplan–Meier survival curves reveal the consistent outcomes of the 2YS predictive model on the two different cohorts; the results from the TGCA dataset were consistent with those from the Wake cohort. The 2YS scores-yield classification results were significantly associated with the overall survival of patients, indicating that the 2YS model can be used as an objective and quantitative tool for GBM diagnosis in clinical practice. Notably, the PsP and TTP groups classified based on the clinical criteria showed no significant association with survival (Log-rank *p* = 0.5884, Figure 4a). This may be due to the variability in the grading process by individual physicians based on follow-up imaging. Since two patients in the Wake dataset do not have diagnosis information of PsP or TTP in clinical records, we excluded these two cases and used the rest of the thirty-seven cases for survival analysis based on the criteria of clinical practice (Figure 4a).

**Figure 5:**
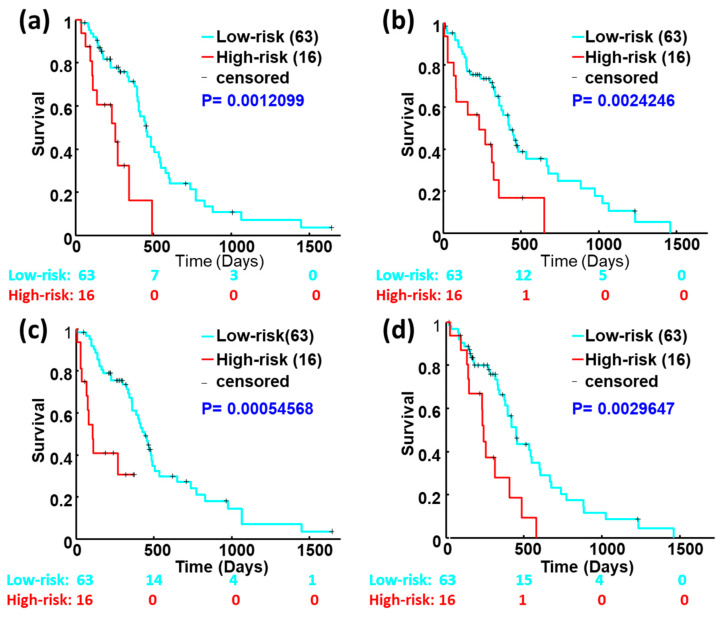
Kaplan–Meier survival analysis of the training and validation datasets using the TCGA-based 2YS model. A total of 158 cases from TCGA were randomly divided into two equal subsets. Each subset was running twice as a testing and validation sample. Panels (**a**,**b**) show the survival analysis results from subset A as training and validation samples. Panels (**c**,**d**) show the survival analysis results from subset B as training and validation samples, respectively.

To further verify the effectiveness of our model, we trained and validated the model using 158 patients’ data from TCGA. The TCGA data were randomly divided into two equal subsets. One subset was used to build the 2YS model and another set was used to validate it. Each subset was running twice as testing and validation samples, respectively. As shown in Figure 5, the low- and high-risk groups from the training dataset or the validation dataset display a significant survival difference (Log-rank *p* < 0.01). These results confirmed the predictive effectiveness of our proposed 2YS prognostic model.

### 3.4. Relevance between Clinical Characteristics and the 2YS Score

To reveal the correlation between clinical factors and the 2YS score, we first used the 2YS score to classify the patients into low- and high-risk groups for the Wake and TCGA cohorts and then performed the Kolmogorov–Simonov test on continuous variables and a Fisher exact test on discrete variables between the two groups, as shown in Table 1. We investigated the association of clinical factors of the Wake dataset with patients’ survival, including age, gender, race, resection type and MGMT. Only the resection type was slightly associated with 2YS scores (Log-rank *p* < 0.03). We also analyzed the clinical variables of the TCGA dataset, including age, gender, race, resection type, ethnicity, KPS, IDH1, MGMT, normal cells ratio, and tumor cells ratio. The IDH1 mutation status was significantly associated with the risk groups (Log-rank *p*-value of 0.0001). Previous studies have shown that IDH1 mutation was a potential biomarker for GBM [40,41]. For example, Moteqi et al. found that IDH1 mutation was associated with PsP and TTP [14].

We assessed the prognostic performance of the 2YS score in the context of other known prognostic factors using the multivariate Cox proportional hazards model, as shown in Table 2. The 2YS score yielded from the Wake dataset was significantly associated with patient survival (Table 2A). We used the multivariate Cox proportional hazards model to test whether individual clinical factors (gender, age, race, surgery type, and MGMT) from the Wake dataset could predict patients’ survival and found that their predictive ability was low. We also constructed a multivariate Cox proportional hazards model using the TCGA dataset (Table 2B). The 2YS score from this model was significantly associated with the overall survival, while most of the other clinical factors, including gender, age, KPS, and normal cell ratio, were not or were marginally correlated with patients’ survival. The tumor cell ratio was the only clinical factor significantly associated with patients’ survival (*p*-value = 0.038).

### 3.5. Assessing the Significance of Selected Genes

To evaluate the significance of gene features in the 2YS model, we performed a bootstrap analysis with 1000 bootstrap iterations on the TCGA dataset. The 95% CIs of the coefficient estimates of the 2YS model for each gene’s expression value were yielded. We identified 12 out of 23 features as the significant features in the 2YS model, as shown in Table 3. Most of these genes have been previously reported to participate in critical biological processes related to the cell cycle and DNA repair. In particular, IRF9 is involved in a series of biological pathways directly or indirectly related to cell proliferation, apoptosis, and innate immunity [42,43,44,45,46,47,48] and has been identified as a potential biomarker for distinguishing PsP and TTP in our previous radiogenomics study [39]. Functional analyses of these genes (Supplementary Materials) indicated that IRF9, MGLL, and OAS1/3 interact with IFNβ and interferon α to exert inflammation functions, while XRCC1, TCTN2, and FOXJ2 act synergistically with TP53 and ADAMTS8 in the regulation of cell cycle-related processes. MGLL, which transcribes the MAGL protein, is also related to the self-renewal ability and tumorigenicity of cancer stem cells (CSCs) [49]. CSCs are an important mechanism of GBM radioresistance.

## 4. Discussion

In this study, we aimed to develop a prognostic model for evaluating the tumor progression potential of GBM patients following standard therapies. To achieve this purpose, we applied a dictionary learning scheme to obtain imaging features of GBM patients with PsP or TTP from the Wake dataset. Based on these radiographic features, we conducted radiogenomics analysis to identify the significantly associated genes. These significant genes were used as features to construct a 2YS logistic regression model. GBM patients were classified into low- and high-survival risk groups based on the individual 2YS scores derived from this model. We tested our model using an independent TCGA dataset and found that 2YS scores were significantly associated with the patients’ overall survival. To further verify the effectiveness of our model, the TCGA dataset was randomly divided into two cohorts. Each cohort was split into training and validating groups. Our results show that the training and validation datasets displayed a significant survival difference (Log-rank *p* < 0.01), confirming the effectiveness and robustness of our proposed 2YS model in predicting the clinical outcomes of GBM patients after standard therapies. However, most of the other clinical factors, including gender, age, KPS, and normal cell ratio, were not or were marginally correlated with patients’ survival.

Most of the existing work involved in quantitative imaging has required the interest of area (ROI) determination of lesions through the laborious outline and semi-/automated segmentation by algorithms, followed by the measurement of expert predefined features, such as size, color, shape, and texture, for describing lesion characteristics. In the case of our work, the ROI determination of GBM on DTI is a tough task due to the low imaging quality [35], although DTI has the potential to differentiate the tumors and brain tissues with treatment effects [33,34]. After the training of the dictionary learning model using expert-derived label annotations, our feature extraction and selection were achieved without relying on segmentation and manual steps, which greatly increases its scalability. Furthermore, the conventional predefined descriptors may be unqualified for completely characterizing the tumors. In contrast, our previously developed classification-oriented dictionary learning scheme provides the specific and common features for different treatment results, enabling the identification of imaging features related to the clinical treatment progression [35].

In recent years, radiogenomics has emerged as a new research field for discovering the relationship between the imaging and genetic profiles of patients, with the purpose of better understanding and promoting the research for cancer treatment [29]. For example, the association map, determined by the radiogenomics study, can discover the specific genetics underlying the imaging characteristics [50] and construct the predictive radiographic signatures [51]. In this study, we conducted a radiogenomics study to explore the relationship between the DTI phenotype and genotype (i.e., gene expression) for the determination of significant genes with our previously developed low-rank sparse regression model. Through this radiogenomics study, these identified significant genes reflect the development of PsP and TTP well, since they were highly associated with the imaging features, and these imaging features from the classification-oriented dictionary learning method could provide a good classification performance for PsP and TTP (Figure 2).

We have further documented the clinical relevance of these significant genes by a series of subsequent functional analyses. Specifically, after obtaining the 23 significant genes from the radiogenomics study, the IPA (Ingenuity Pathway Analysis) software was employed to explore the biological functions of these genes, including critical signaling pathways they participate in, diseases or functions they are associated with, and biological networks they are involved in. The canonical pathway analysis tool was used to determine the most significantly affected pathways in the context of established signaling and metabolic pathways and determine which pathways overlap based on molecules in common. The enrichment significance and candidate gene coverage profile are calculated. The disease/function annotation tool provides details associated with the disease or biological function such as related molecules or known drug targets. The network analysis tool was applied to build and explore transcriptional networks, microRNA-mRNA target networks, phosphorylation cascades, and protein–protein or protein–DNA interaction networks. As shown in the Supplementary Materials, Figure S1 illustrates the top-ranked signaling pathways associated with the 23 candidate genes. IRF9 is involved in the interferon signaling pathway, while XRCC1 takes part in both the BER (base excision repair) and DDSB (DNA double-strand break) repair pathways. Table S3 lists the most relevant (*p* < 10^−3^) diseases and biological functions in which these genes are implicated. IRF9 and MGLL are involved in the inflammation process, and ERCC1 and XRCC1 are associated with the cell cycle (G2/M) process. Figure S4 shows the interaction network between the significant genes and other related molecules. IRF9 and OAS1/3 interact with the inflammatory genes IFNb and interferon α and are also indirectly associated with histone 3 and 4, while XRCC1, TCTN2, and FOXJ2 synergistically interact with TP53 and ADAMTS8 to exert their cell cycle-related functions. Genes such as OAS3, TULP3, IFI6, MGLL, BAZ2A, ERCC1, OAS1, and RAB8A have also shown dysregulation in different cell types of drug-resistant groups in other types of cancer [52]. MGLL, which transcribes the MAGL protein, is related to CSCs. The overexpression of MAGL could enhance the self-renewal ability and tumorigenicity of CSCs [49]. A previous study reported that glioma stem cells are resistant to radiation and are directly correlated with patients’ outcomes [53]. Our results further demonstrated that CSCs play a role in GBM radioresistance.

Despite the annotated merits, the radiogenomics approach has the other side of a coin, i.e., integrating phenotype and genotype data from each patient objectively and inevitably limiting the sample size for significant genes determination, which is also a probable imperfection of this study. It is a challenging task to simultaneously collect all types of datasets from individual patients, including DTI, gene expression, and clinical records, because these datasets were generated for clinical purposes and not specially designed for academic research. Furthermore, TCGA and the Cancer Imaging Archive (TCIA) do not include clinical records regarding treatment results (i.e., the triage of patients with standard therapies) and DTI. Therefore, the TCGA dataset in this study could not be used to perform the radiogenomics study for exploring the significant gense, but it can be employed for the validation of the 2YS model. However, owing to the successful identification of significant genes in the Wake dataset, we have reached a good result from a small set of training samples and independently validated the model on the TCGA dataset with desirable performance. This suggests that we have derived a powerful prognostic model. Before the translation of this system for use in clinical practice, this model should be trained on the dataset from diverse cohorts and tested on additional independent cohorts to better evaluate the model’s generalizability.

Figure 4a presents an unexpected and intriguing result: there is no significant survival difference with 0.5884 of the Log-rank *p* value between the PsP and TTP groups by clinical diagnosis from the Wake dataset. This result is not surprising, since it is not an isolated case. For example, recently, several works uncovered no significant association with survival for different stages of breast cancer on some cohorts by current clinical criteria [30,54]. This motivated us to explore a novel prognostic marker for the stratification of GBM patients into different risk groups with significant survival associations. There are several potential factors causing this abnormal phenomenon (Figure 4a). First, the clinical practice is to perform follow-up MRI examinations based on the morphological changes in lesions. It usually takes several months to make the final diagnosis and causes an adverse effect on the clinical management of patients. Second, the clinical diagnosis criterion for PsP and TTP is defined based on the phenotype progression, which is unable to recover the disease’s essential progression comprehensively. Pathological confirmation presents a reliable diagnosis, whereas brain tumor biopsies are not usually applied in clinical practice due to their invasiveness, increasing the risk for subsequent therapy. Thus, the imaging-based diagnosis may lead to an insignificant survival difference between the two entities. Finally, the limited sample size may result in outcome bias. We will further collect cases to verify this discovery, although it will take a long time. In our previous work, we have tried to overcome the critical delay of diagnosis (i.e., the first problem mentioned above) through developing a computer-aided diagnosis system based on DTI [35]. To deal with the second issue noted above, we integrated imaging features and gene expressions to determine the 23 significant genes as features for the construction of a predictive survival model, which was significantly associated with survival.

From the view of clinicians, our 2YS score provides an innovative diagnosis index, which bridges the radiogenomic biomarkers and clinical survival status. Physicians can utilize the 2YS score to give reliable estimations of the GBM progression risk. Compared to current medical judgments, the 2YS score only needs partial retrospective information on patients’ genomic and radiographic records. In this way, long follow-up processes and invasive pathologic detections can be avoided. Thus, the 2YS score can be considered as an actionable clinical assistance index for judging GBM prognostics. Furthermore, the 2YS score is verified through the statistical association with some known omics biomarkers. In Table 1, “IDH1 mutation” is highly associated with high and low risks of the 2YS Score (*p*-values < 0.05). We discovered that “tumor cells” own a similar GBM survival risk as the 2YS score in Table 2 (*p*-value: 0.0383). In sum, our proposed 2YS score proves to be clinically meaningful and an important diagnostic complement for GBM physicians.

## Figures and Tables

**Table 1:** Patient characteristics categorized by 2YS score.

Patients’ Characteristics		2YS Score		*P*-Value
		Low-Risk	High-Risk	
**Wake Dataset**				
n		26	13	
Age [mean IQR]		58.4 [49.2 69.8]	60.6 [51.7 68.5]	0.869
Gender (%)	Male	13 (50%)	6 (46.2%)	1
	Female	13 (50%)	7 (53.8%)	
Race (%)	White	13 (50%)	7 (53.8%)	1
	Black or African American	13 (50%)	6 (46.2%)	
	Asian	0	0	
Resection Type (%)	Tumor Resection	25 (96.2%)	12 (92.3%)	0.03452
	Biopsy	1 (3.8%)	1 (7.7%)	
MGMT (%)	Hypermethylation	15 (60%)	9 (69.2%)	0.7281
	Hypomethylation	10 (40%)	4 (30.8%)	
**TCGA Dataset**				
N		91	67	
Age [mean IQR]		61.3 [54 72]	59.4 [50 70]	0.1592
Gender (%)	Male	54 (59.3%)	48 (71.6%)	0.131
	Female	37 (40.7%)	19 (28.4%)	
Race (%)	White	82 (91.1%)	56 (84.9%)	0.104
	Black or African American	4 (4.4%)	8 (12.1%)	
	Asian	2 (4.4%)	4 (3.0%)	
Ethnicity	Hispanic or Latino	0 (0%)	1 (2.0%)	0.382
	Not Hispanic or Latino	79 (100%)	48 (98.0%)	
Resection Type	Tumor Resection	81 (89.0%)	59 (88.1%)	1
	Biopsy	10 (11.0%)	8 (11.9%)	
KPS (%)	<=60	21 (29.2%)	10 (21.3%)	0.673
	70	3 (4.2%)	2 (4.2%)	
	80	40 (55.6%)	26 (55.3%)	
	90	2 (2.8%)	3 (6.4%)	
	100	6 (8.2%)	6 (12.8)	
IDH1 (%)	Mutation	10 (11.0%)	0 (0%)	0.0001
	Normal	81 (89.0%)	67 (100%)	
MGMT (%)	Hypermethylation	14 (41.2%)	7 (30.4%)	0.5765
	Hypomethylation	20 (58.8%)	16 (69.6%)	
Normal cells [mean, IQR]		3.02% [0, 0]	0.5% [0, 0]	0.965
Tumor cells [mean, IQR]		85.9% [80%, 98%]	84.7% [80%, 99%]	0.628

**Table 2:** Multivariate Cox Proportional Hazards Model for Overall Survival.

	Exp (Coef)	Exp (-Coef)	Low 95%	Upper 95%	*p*
**A**. Multivariate Cox regression (Wake Dataset, *n* = 38)					
2YS model score	3.1067	0.3219	2.6518	4.8614	0.0166
Gender	0.9879	1.2016	0.9673	1.0074	0.6874
Age	0.8322	1.0122	0.6207	0.9934	0.4716
Race	0.3649	2.7406	0.2380	0.4412	0.0575
Surgery Type	0.1879	5.3219	0.1476	0.2317	0.0543
MGMT	0.5124	1.9518	0.0317	4.0883	0.8744
**B**. Multivariate Cox regression (TCGA, *n* = 117)					
2YS model score	1.6752	0.5970	1.4674	1.9305	0.0087
Gender	1.5819	0.6322	1.3734	1.8748	0.0598
Age	1.0018	0.9982	0.9974	1.0063	0.8322
KPS	1.0029	0.9971	0.9979	1.0082	0.7524
Normal cells	1.0782	0.9275	1.0513	1.1206	0.0761
Tumor cells	1.0126	0.9876	1.0097	1.0166	0.0383

**Table 3:** Identification of significant gene features in the 2YS model by bootstrap analysis.

	Feature (Genes)	95% Low	95% High
1	IRF9	0.5721	0.8568
2	OAS3	−0.9602	−0.3276
3	TULP3	−0.8305	−0.3923
4	IFI6	−0.6036	−0.0959
5	MGLL	0.4811	1.4234
6	BAZ2A	0.2777	0.6136
7	ERCC1	−0.9589	−0.0290
8	OAS1	0.0451	0.7442
9	FOXJ2	−0.7236	−0.0889
10	RAB8A	−0.9366	−0.2549
11	TAB3	0.1637	0.8704
12	TCTN2	0.0467	0.8706

## Data Availability

Requests for the data information should be directed to Xiaobo Zhou (xiaobo.zhou@uth.tmc.edu).

## Supplementary Materials

The following supporting information can be downloaded at: https://www.mdpi.com/article/10.3390/genes15060718/s1.
