# Supplementary material for: Radiogenomics-Based Risk Prediction of Glioblastoma Multiforme with Clinical Relevance
Source: Genes (Basel). 2024 Jun 1;15(6):718. doi: 10.3390/genes15060718 (PMC11202835; doi:10.3390/genes15060718)
Supplement: Supplementary file 1 [file genes-15-00718-s001.zip › genes-2986449-supplementary.pdf]

Supplemental Figures and Tables

Figure legends:

Figure S1. JAK-STAT pathway for type I IFN, retrieved and summarized from previous studies.

Figure S2. Top-ranked Canonical Pathways associated with the **33 candidate genes selected by radiogenomics**. Canonical pathways are ordered by the *p*-values ($p<0.05$).

Figure S3. Top-ranked Canonical Pathways associated with the 119 differentially expressed genes. Top-ranked Canonical Pathways were highlighted red in (b) with p<0.05.

Figure S4. The ROC curve for the classification of the 119 differentially expressed genes as features. The SVM classifier and fivefold cross-validation were applied in this study.

Figure S5. The gene overlaps among candidate genes from different coverage rates *P* and genes from top-ranked canonical pathways (i.e., C_Pathway). The coverage rate *P* was defined as the occurrence frequency of genes on the lists of weight ranker top 50 in 27 parameter combinations. *P*_100, *P*_90 and *P*_80 mean 100%, 90% and 80% of coverage *P*, respectively. Experiments with six feature sets: (a) 225 image features; (b) 50 image features used in clinic practices; (c) 50 image features randomly selected from (a); (d)-(f) 20 image features randomly selected from the 50 clinical features in (b) for three times.

Figure S6. Boxplots of gene methylation, promoter methylation with 1k, 2k, and 3k window of TSS from 1 to 4 columns, respectively, for IRF9 (1st row), XRCC1(2nd row), and MGMT (3rd row).

Figure S7. Demonstration of PsP and TTP based on longitudinal MRI. All of the images are from TCIA. The sample ID for the top row images is TCGA-06-0185, and the bottom row is TCGA-06-1084.

Figure S8. The flowchart of the segmentation of tumor regions (i.e., enhanced region and necrosis regions).

Table legends:

Table S1. Summary of all the features extracted from the longitudinal MRI

Table S2. Top 119 significant genes screened by the Wilcoxon rank sum test with P-value of 0.005.

Table S3. The candidate genes selected from three representative feature sets of 255 features, 50 clinical features and 20 clinical features (corresponding to the Fig. S5 (a), (b) and (d), respectively). *P_100, P_90 and P_80* mean 100%, 90% and 80% of coverage *P*, respectively. The genes from top ranked canonical pathways were in the right column.

Table S4. Predication of MGMT methylation status in previous studies

Table S5. Gene expressions of IRF9 and XRCC1 in the samples from TCGA. The sample type, i.e., pseudoprogression (PsP) or true tumor progression (TTP), is determined by the longitudinal MRI from TCIA.

Comparison with classic scheme (i.e. directly using the sample labels for marker identification)

We have compared our approach with the classic scheme (i.e. directly using the sample labels for marker identification), as shown in supplementary Fig. S2 and S3. Actually, our approach also adopted the phenotype labels. Specifically, in the first step, we compared the gene expression levels in the PsP and TTP groups to obtain the differentially expressed genes. We initially identified 119 genes using the Wilcoxon rank sum test with $p<0.005$. After that, we selected 33 candidate genes using associations study between imaging features and gene expression profiles with two advantages: 1) the imaging features were extracted from tumors along the longitudinal MRI and provided diagnostic information of PsP and TTP. Thus, the 33 candidate genes were confirmed to be associated with the development of PsP and TTP. 2) The association study can narrow down candidate genes from 119 to 33, which improves the efficiency of the biological relevance analysis. In summary, the association study based on the differentially expressed genes facilitates the biological analysis in efficiently identifying most relevant signaling pathways.

To illustrate the advantage of our approach, we conducted the comparison experiments (Fig. S2 and S3). First, we only used the phenotype labels to obtain 119 differentially expressed genes using the Wilcoxon rank sum test with $p<0.005$. We then performed the biological analysis for the 119 genes without the association study. Top-ranked Canonical Pathways and their genes by IPA were shown in Fig. S3. Obviously, the XRCC1 and its pathways, such as BER pathway, DNA Double-strand Break Repair by Non-Homologous End Joining, and DNA damage-induced 14-3-3σsignaling, in the top-ranked lists of our study (Association study, Fig. S2) were absent from the significant lists of the classic scheme (Fig. S3 (a)). The p-value of these XRCC1 pathways from the classic scheme was slightly greater than 0.05 (Fig. S3 (b)). Other pathways in the top-ranked list, such as the Role of RIG1-like Receptors in Antiviral innate immunity, the Role of BRCA1 in DAN damage response, and the role of pattern recognition receptors in recognition of bacteria and viruses, were not directly related to cancer development. This result indicates

that differentially expressed genes without relation to the PsP and TTP may confound the biological analysis. Therefore, the association study can not only confirm the candidate genes with relation to the development of PsP and TTP but also narrow down the candidate genes, thereby ensuring the real significant genes stand out in the biological analysis.

Effect of morphological features with different size

To illustrate the effect of features, we conducted comparisons of six feature sets with different sizes, as shown in Fig. S5. The six feature sets were 255 morphological features, 50 clinical morphological features, 50 features randomly selected from our 255 features, and 20 features randomly selected from the 50 clinical features for three times, corresponding to Figure S5 (a)-(f), respectively. Specifically, we selected 50 clinical morphological features from 255 features, corresponding to the VASARI, which offers a set of well-defined terms that describe the GBM tumors[1]. The 50 clinical morphological features contain the major/minor axis length of the enhanced region, the thickness of enhancing margin, tumor volume, proportion enhancing and proportion necrosis, etc. In each experiment of the individual feature set, there were 27 parameter combinations for our model. We defined the coverage rate *P* as the occurrence frequency of genes on the lists of weight ranked top 50 in 27 parameter combinations. As a result, we can obtain three different sets of candidate genes with 80%, 90% and 100% of coverage *P* for the individual feature set. Results from three representative feature sets of 255 features, 50 clinical features, and 20 clinical features (corresponding to the Fig. S5 (a), (b) and (d), respectively) were shown in Table S3. Then, the candidate genes identified by coverage $P=0.8$ were used for pathway analysis using IPA. Not surprisingly, the genes from the top-ranked canonical pathways from six feature sets, called pathway genes, were same, as shown in the right column of Table S3. Fig. S5 shows the gene overlap among candidate genes from different coverage *P* and pathway genes. Obviously, when the feature number is 20 or 50, there is no overlap between candidate genes from $P=100\%$ and pathway genes. Conversely, our model with 255 features yielded the biomarkers, i.e., IRF9 and XRCC1, in all of the three coverage rates. These results indicate that the performance of our model with 255 features is more robust than that with 50 or 20 features.

Reference:

1 Thomas Jefferson University hospital, “VASARI MRI visual feature guide” (2010)

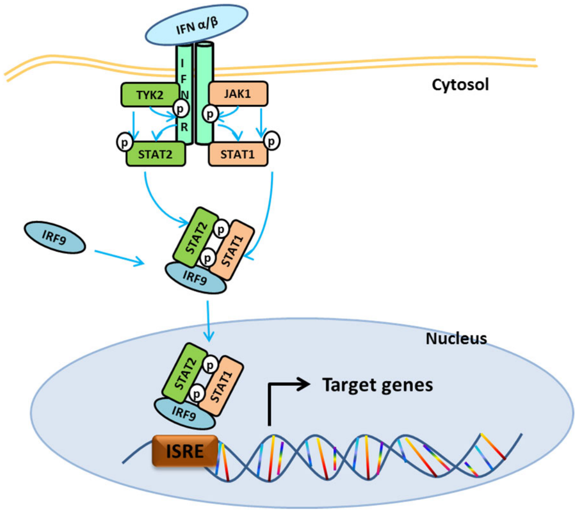

Figure S1. JAK-STAT pathway for type I IFN, retrieved and summarized from previous studies.

(a)

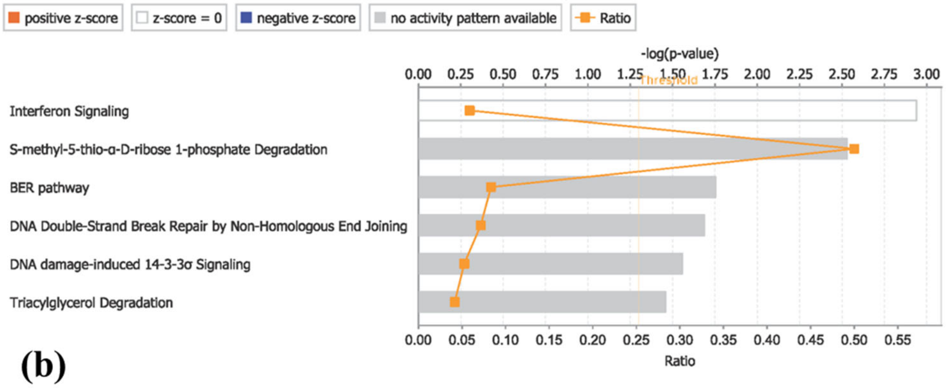

(b)

Ingenuity Canonical Pathways	-log(p-value)	Ratio	Molecules
Interferon Signaling	2.94E00	5.88E-02	IRF9,IFIT3
S-methyl-5-thio-α-D-ribose 1-phosphate Degradation	2.53E00	5E-01	MRI1
BER pathway	1.76E00	8.33E-02	XRCC1
DNA Double-Strand Break Repair by Non-Homologous End Joining	1.69E00	7.14E-02	XRCC1
DNA damage-induced 14-3-3σ Signaling	1.56E00	5.26E-02	RAD9A
Triacylglycerol Degradation	1.46E00	4.17E-02	MGLL

Figure S2. Top-ranked Canonical Pathways associated with the **33 candidate genes selected by radiogenomics**. Canonical pathways are ordered by the *p*-values ($p<0.05$).

(a)

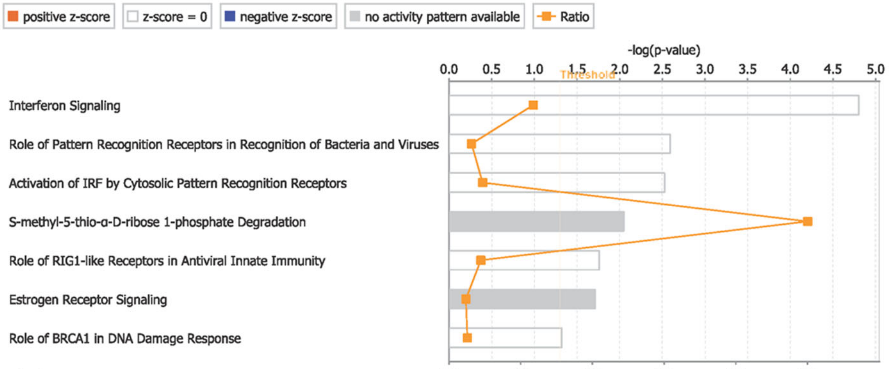

(b)

Ingenuity Canonical Pathways	-log(p-value)	Ratio	Molecules
Interferon Signaling	4.8E00	1.18E-01	OAS1,IRF9,IFIT1,IFIT3
Role of Pattern Recognition Receptors in Recognition of Bacteria and Viruses	2.59E00	3.15E-02	OAS1,OAS3,OAS2,DDX58
Activation of IRF by Cytosolic Pattern Recognition Receptors	2.53E00	4.69E-02	DHX58,IRF9,DDX58
S-methyl-5-thio-α-D-ribose 1-phosphate Degradation	2.05E00	5E-01	MRI1
Role of RIG1-like Receptors in Antiviral Innate Immunity	1.76E00	4.44E-02	DHX58,DDX58
Estrogen Receptor Signaling	1.71E00	2.36E-02	MED31,MED12,TAF1
Role of BRCA1 in DNA Damage Response	1.32E00	2.56E-02	FAM175A,ATF1
BER pathway	1.28E00	8.33E-02	XRCC1
DNA Double-Strand Break Repair by Non-Homologous End Joining	1.22E00	7.14E-02	XRCC1
DNA damage-induced 14-3-3σ Signaling	1.09E00	5.26E-02	RAD9A
Triacylglycerol Degradation	9.91E-01	4.17E-02	MGLL

Figure S3. Top-ranked Canonical Pathways associated with the **119 differentially expressed genes**. Top-ranked Canonical Pathways were highlighted red in (b) with $p<0.05$.

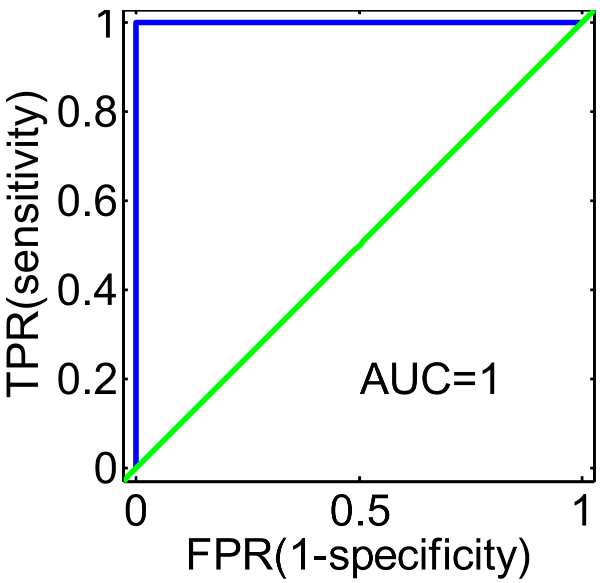

Figure S4. The ROC curve for the classification of the 119 differentially expressed genes as features. The SVM classifier and fivefold cross-validation were applied in this study. $sensitivity = TP/(TP + FN)$ and $specificity = TN/(FP + TN)$, where TP, FP, FN, and TN refer to true positive, false positive, false negative, and true negative, respectively.

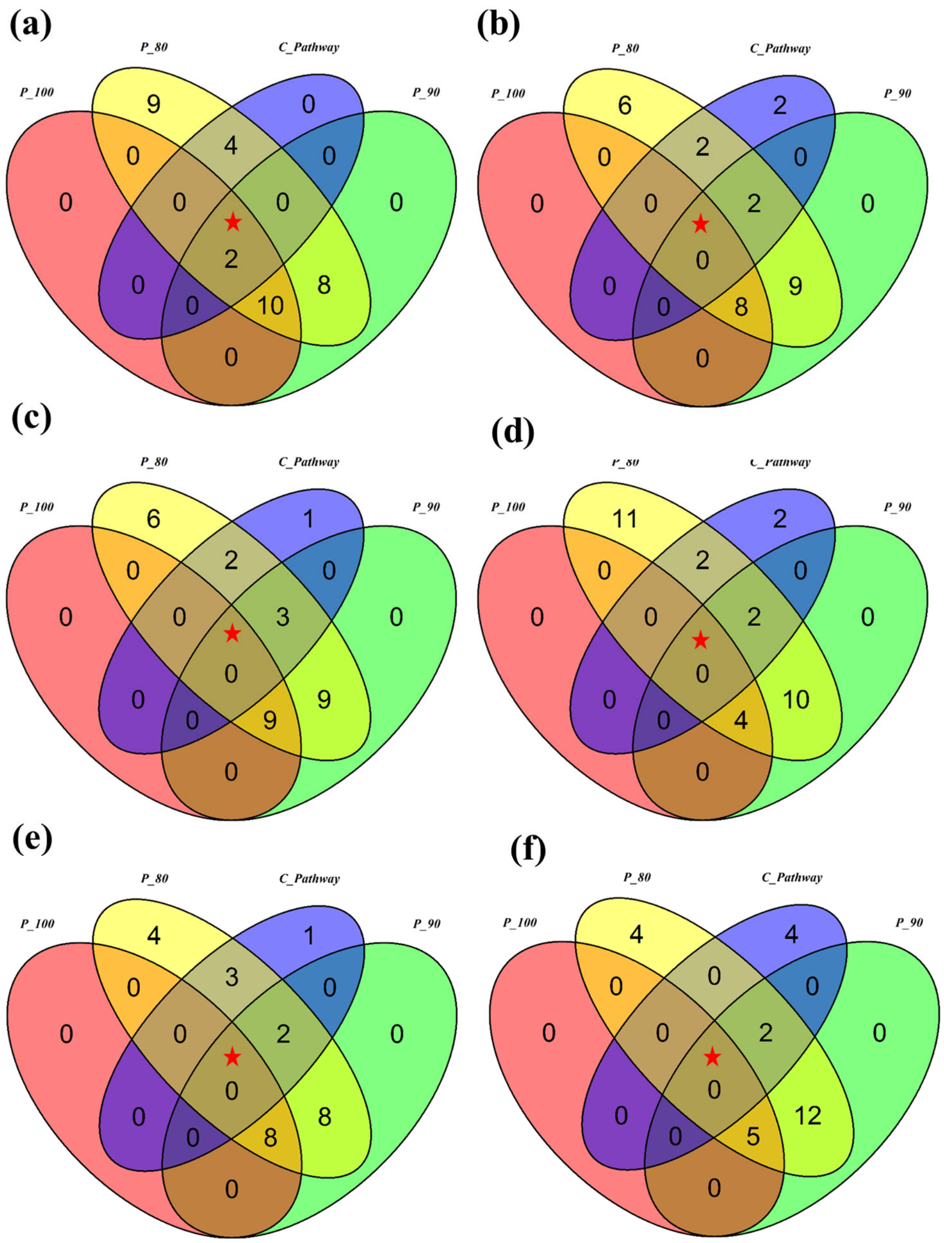

Figure S5. The gene overlaps among candidate genes from different coverage rates *P* and genes from top-ranked canonical pathways (i.e., *C_Pathway*). The coverage rate *P* was defined as the occurrence frequency of genes on the lists of weight ranker top 50 in 27 parameter combinations. *P_100, P_90 and P_80* mean 100%, 90% and 80% of coverage *P*, respectively. Experiments with six feature sets: (a) 225 image features; (b) 50 image features used in clinic practices; (c) 50 image features randomly selected from (a); (d)-(f) 20 image features randomly selected from the 50 clinical features in (b) for three times.

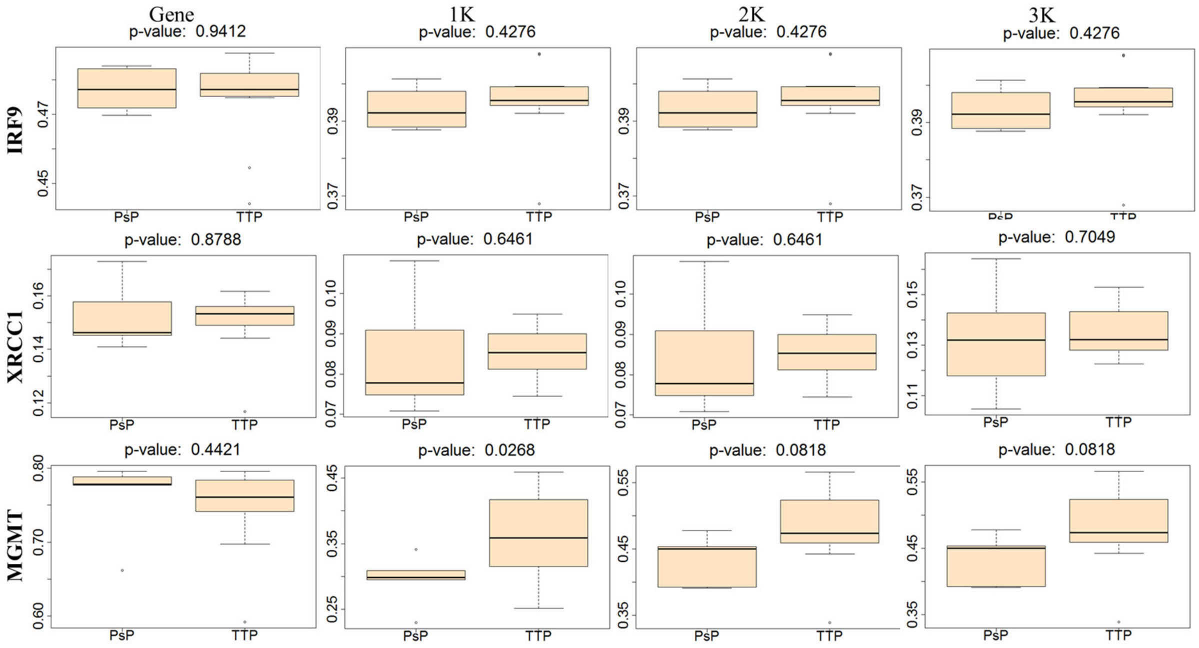

Figure S6. Boxplots of gene methylation, promoter methylation with 1k, 2k, and 3k window of TSS from 1 to 4 columns, respectively, for IRF9 (1st row), XRCC1(2nd row), and MGMT (3rd row).

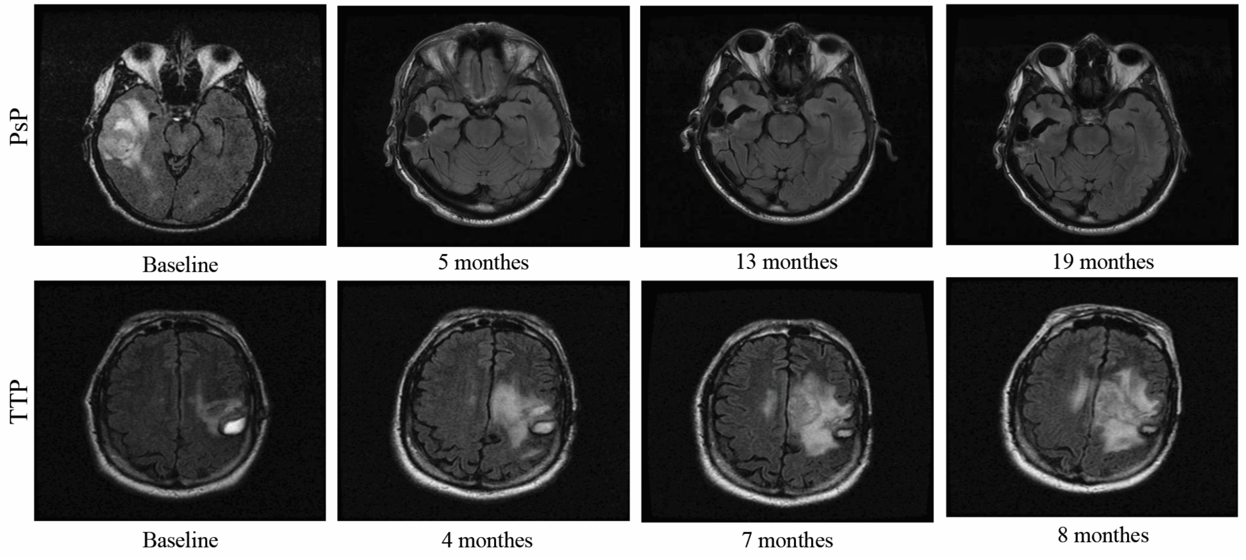

Figure S7. Demonstration of PsP and TTP based on longitudinal MRI. All of the images are from TCIA. The sample ID for the top row images is TCGA-06-0185, and the bottom row is TCGA-06-1084.

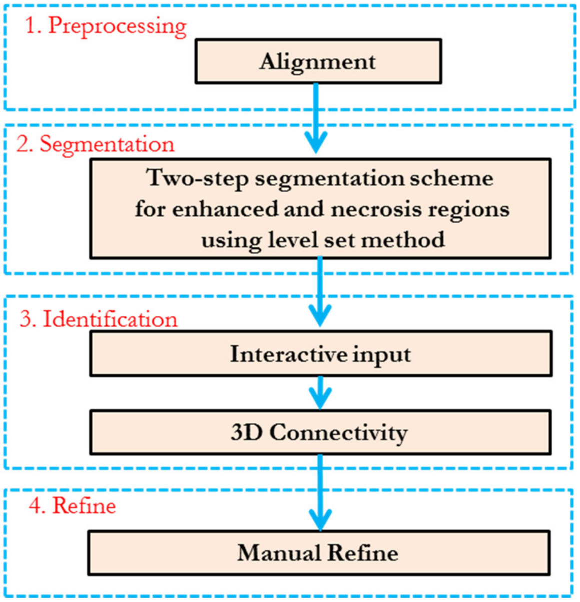

Figure S8. The flowchart of the segmentation of tumor regions (i.e., enhanced region and necrosis regions).

Supplemental Tables

Table S1. Summary of all the features extracted from the longitudinal MRI

**Features extraction from the enhanced region (1-80: total sum, representative case, mean, max and min value)**	
Total sum of features from slices in a case 1-16	
1	Area ration of enhanced region and necrosis region
2	Area of enhanced region
3	Length of enhanced region
4	Width of enhanced region
5	Radius of the enhanced region considered as circle region
6	Regions number in the enhanced region
7	Maximum area in the enhanced regions
8	Perimeter of the enhanced region with maximum area
9	Eccentricity of the enhanced region with maximum area
10	Compactness of the enhanced region with maximum area
11	Sphericity of the enhanced region with maximum area
12	Orientation of the enhanced region with maximum area
13	Bounding box area of the enhanced region with maximum area
14	Major axis length of the enhanced region with maximum area
15	Minor axis length of the enhanced region with maximum area
16	Solidity of the enhanced region with maximum area
Feature from the slice with biggest tumor area in a case 17-32	
17	Area ration of enhanced region and necrosis region
18	Area of enhanced region
19	Length of enhanced region
20	Width of enhanced region
21	Radius of the enhanced region considered as circle region
22	Regions number in the enhanced region
23	Maximum area in the enhanced regions
24	Perimeter of the enhanced region with maximum area
25	Eccentricity of the enhanced region with maximum area
26	Compactness of the enhanced region with maximum area
27	Sphericity of the enhanced region with maximum area
28	Orientation of the enhanced region with maximum area
29	Bounding box area of the enhanced region with maximum area
30	Major axis length of the enhanced region with maximum area
31	Minor axis length of the enhanced region with maximum area
32	Solidity of the enhanced region with maximum area
Mean value of features extracted from a case 33-48	
33	Area ration of enhanced region and necrosis region
34	Area of enhanced region
35	Length of enhanced region
36	Width of enhanced region
37	Radius of the enhanced region considered as circle region
38	Regions number in the enhanced region
39	Maximum area in the enhanced regions
40	Perimeter of the enhanced region with maximum area
41	Eccentricity of the enhanced region with maximum area
42	Compactness of the enhanced region with maximum area
43	Sphericity of the enhanced region with maximum area
44	Orientation of the enhanced region with maximum area
45	Bounding box area of the enhanced region with maximum area
46	Major axis length of the enhanced region with maximum area
47	Minor axis length of the enhanced region with maximum area
48	Solidity of the enhanced region with maximum area

Maximum value of features extracted from a case 49-64

49	Area ration of enhanced region and necrosis region
50	Area of enhanced region
51	Length of enhanced region
52	Width of enhanced region
53	Radius of the enhanced region considered as circle region
54	Regions number in the enhanced region
55	Maximum area in the enhanced regions
56	Perimeter of the enhanced region with maximum area
57	Eccentricity of the enhanced region with maximum area
58	Compactness of the enhanced region with maximum area
59	Sphericity of the enhanced region with maximum area
60	Orientation of the enhanced region with maximum area
61	Bounding box area of the enhanced region with maximum area
62	Major axis length of the enhanced region with maximum area
63	Minor axis length of the enhanced region with maximum area
64	Solidity of the enhanced region with maximum area

Minimum value of features extracted from a case 65-80

65	Area ration of enhanced region and necrosis region
66	Area of enhanced region
67	Length of enhanced region
68	Width of enhanced region
69	Radius of the enhanced region considered as circle region
70	Regions number in the enhanced region
71	Maximum area in the enhanced regions
72	Perimeter of the enhanced region with maximum area
73	Eccentricity of the enhanced region with maximum area
74	Compactness of the enhanced region with maximum area
75	Sphericity of the enhanced region with maximum area
76	Orientation of the enhanced region with maximum area
77	Bounding box area of the enhanced region with maximum area
78	Major axis length of the enhanced region with maximum area
79	Minor axis length of the enhanced region with maximum area
80	Solidity of the enhanced region with maximum area

Features extraction from the necrosis region (81-150: total sum, representative case, mean, max and min value)

Total sum of features from slices in a case 81-94

81	Area ration of enhanced region and necrosis region
82	Area of necrosis region
83	Radius of the necrosis region considered as circle region
84	Regions number in the necrosis region
85	Maximum area in the necrosis regions
86	Perimeter of the necrosis region with maximum area
87	Eccentricity of the necrosis region with maximum area
88	Compactness of the necrosis region with maximum area
89	Sphericity of the necrosis region with maximum area
90	Orientation of the necrosis region with maximum area
91	Bounding box area of the necrosis region with maximum area
92	Major axis length of the necrosis region with maximum area
93	Minor axis length of the necrosis region with maximum area
94	Solidity of the necrosis region with maximum area

Feature from the slice with biggest tumor area in a case 95-108

95	Area ration of enhanced region and necrosis region
96	Area of necrosis region
97	Radius of the necrosis region considered as circle region
98	Regions number in the necrosis region

99	Maximum area in the necrosis regions
100	Perimeter of the necrosis region with maximum area
101	Eccentricity of the necrosis region with maximum area
102	Compactness of the necrosis region with maximum area
103	Sphericity of the necrosis region with maximum area
104	Orientation of the necrosis region with maximum area
105	Bounding box area of the necrosis region with maximum area
106	Major axis length of the necrosis region with maximum area
107	Minor axis length of the necrosis region with maximum area
108	Solidity of the necrosis region with maximum area
Mean value of features extracted from a case 109-122	
109	Area ration of enhanced region and necrosis region
110	Area of necrosis region
111	Radius of the necrosis region considered as circle region
112	Regions number in the necrosis region
113	Maximum area in the necrosis regions
114	Perimeter of the necrosis region with maximum area
115	Eccentricity of the necrosis region with maximum area
116	Compactness of the necrosis region with maximum area
117	Sphericity of the necrosis region with maximum area
118	Orientation of the necrosis region with maximum area
119	Bounding box area of the necrosis region with maximum area
120	Major axis length of the necrosis region with maximum area
121	Minor axis length of the necrosis region with maximum area
122	Solidity of the necrosis region with maximum area
Maximum value of features extracted from a case 123-136	
123	Area ration of enhanced region and necrosis region
124	Area of necrosis region
125	Radius of the necrosis region considered as circle region
126	Regions number in the necrosis region
127	Maximum area in the necrosis regions
128	Perimeter of the necrosis region with maximum area
129	Eccentricity of the necrosis region with maximum area
130	Compactness of the necrosis region with maximum area
131	Sphericity of the necrosis region with maximum area
132	Orientation of the necrosis region with maximum area
133	Bounding box area of the necrosis region with maximum area
134	Major axis length of the necrosis region with maximum area
135	Minor axis length of the necrosis region with maximum area
136	Solidity of the necrosis region with maximum area
Minimum value of features extracted from a case 137-150	
137	Area ration of enhanced region and necrosis region
138	Area of necrosis region
139	Radius of the necrosis region considered as circle region
140	Regions number in the necrosis region
141	Maximum area in the necrosis regions
142	Perimeter of the necrosis region with maximum area
143	Eccentricity of the necrosis region with maximum area
144	Compactness of the necrosis region with maximum area
145	Sphericity of the necrosis region with maximum area
146	Orientation of the necrosis region with maximum area
147	Bounding box area of the necrosis region with maximum area
148	Major axis length of the necrosis region with maximum area
149	Minor axis length of the necrosis region with maximum area
150	Solidity of the necrosis region with maximum area

Features extraction from the tumor region (151-225: total sum, representative case, mean, max and min value)	
Total sum of features from slices in a case 151-165	
151	Area of tumor region
152	x coordinate of centroid point
153	y coordinate of centroid point
154	Radius of the tumor region considered as circle region
155	Regions number in the tumor region
156	Maximum area in the tumor regions
157	Perimeter of the tumor region with maximum area
158	Eccentricity of the tumor region with maximum area
159	Compactness of the tumor region with maximum area
160	Sphericity of the tumor region with maximum area
161	Orientation of the tumor region with maximum area
162	Bounding box area of the tumor region with maximum area
163	Major axis length of the tumor region with maximum area
164	Minor axis length of the tumor region with maximum area
165	Solidity of the tumor region with maximum area
Feature from the slice with biggest tumor area in a case 166-180	
166	Area of tumor region
167	x coordinate of centroid point
168	y coordinate of centroid point
169	Radius of the tumor region considered as circle region
170	Regions number in the tumor region
171	Maximum area in the tumor regions
172	Perimeter of the tumor region with maximum area
173	Eccentricity of the tumor region with maximum area
174	Compactness of the tumor region with maximum area
175	Sphericity of the tumor region with maximum area
176	Orientation of the tumor region with maximum area
177	Bounding box area of the tumor region with maximum area
178	Major axis length of the tumor region with maximum area
179	Minor axis length of the tumor region with maximum area
180	Solidity of the tumor region with maximum area
Mean value of features extracted from a case 181-195	
181	Area of tumor region
182	x coordinate of centroid point
183	y coordinate of centroid point
184	Radius of the tumor region considered as circle region
185	Regions number in the tumor region
186	Maximum area in the tumor regions
187	Perimeter of the tumor region with maximum area
188	Eccentricity of the tumor region with maximum area
189	Compactness of the tumor region with maximum area
190	Sphericity of the tumor region with maximum area
191	Orientation of the tumor region with maximum area
192	Bounding box area of the tumor region with maximum area
193	Major axis length of the tumor region with maximum area
194	Minor axis length of the tumor region with maximum area
195	Solidity of the tumor region with maximum area
Maximum value of features extracted from a case 196-210	
196	Area of tumor region
197	x coordinate of centroid point
198	y coordinate of centroid point
199	Radius of the tumor region considered as circle region
200	Regions number in the tumor region
201	Maximum area in the tumor regions
202	Perimeter of the tumor region with maximum area

203	Eccentricity of the tumor region with maximum area
204	Compactness of the tumor region with maximum area
205	Sphericity of the tumor region with maximum area
206	Orientation of the tumor region with maximum area
207	Bounding box area of the tumor region with maximum area
208	Major axis length of the tumor region with maximum area
209	Minor axis length of the tumor region with maximum area
210	Solidity of the tumor region with maximum area

Maximum value of features extracted from a case 211-225

211	Area of tumor region
212	x coordinate of centroid point
213	y coordinate of centroid point
214	Radius of the tumor region considered as circle region
215	Regions number in the tumor region
216	Maximum area in the tumor regions
217	Perimeter of the tumor region with maximum area
218	Eccentricity of the tumor region with maximum area
219	Compactness of the tumor region with maximum area
220	Sphericity of the tumor region with maximum area
221	Orientation of the tumor region with maximum area
222	Bounding box area of the tumor region with maximum area
223	Major axis length of the tumor region with maximum area
224	Minor axis length of the tumor region with maximum area
225	Solidity of the tumor region with maximum area

Table S2. Top 119 significant genes screened by the Wilcoxon rank sum test with P-value of 0.005.

Rand	SYMBOL	SEQID	P-Value
1	---	---	0.000323
2	AP1M1	NM_001130524	0.000323
3	ARMC9	ENST00000359743	0.000323
4	ATF1	NM_005171	0.000323
5	ATP7A	NM_000052	0.000323
6	C19orf66	ENST00000253110	0.000323
7	CAMK2N2	NM_033259	0.000323
8	FAM123B	NM_152424	0.000323
9	HERC5	NM_016323	0.000323
10	HIST1H4G	NM_003547	0.000323
11	IFI44L	NM_006820	0.000323
12	IFIT3	NM_001031683	0.000323
13	IRF9	NM_006084	0.000323
14	NTNG1	NM_001113226	0.000323
15	OAS3	ENST00000228928	0.000323
16	TULP3	NM_003324	0.000323
17	USP18	ENST00000215794	0.000323
18	---	---	0.000646
19	---	---	0.000646
20	C1GALT1C1	NM_152692	0.000646
21	DHX58	NM_024119	0.000646
22	DMRTA1	NM_022160	0.000646
23	DYNLL2	NM_080677	0.000646
24	HERC6	NM_017912	0.000646
25	TAS2R19	NM_176888	0.000646
26	---	---	0.001293
27	---	---	0.001293
28	---	---	0.001293
29	APBB3	ENST00000357560	0.001293
30	CCDC77	NM_032358	0.001293
31	DDX60	NM_017631	0.001293
32	FAM122C	NM_001170779	0.001293
33	FBXO40	ENST00000338040	0.001293
34	IFI6	NM_002038	0.001293
35	IGF1R	NM_000875	0.001293
36	MDM1	NM_017440	0.001293
37	MED12	NM_005120	0.001293
38	MGLL	ENST00000434178	0.001293
39	MTFMT	NM_139242	0.001293

40	OR10A2	ENST00000307322	0.001293
41	PARP12	ENST00000263549	0.001293
42	PHF11	NM_001040443	0.001293
43	RASGEF1C	ENST00000393371	0.001293
44	U2AF1L4	NM_001040425	0.001293
45	ZNF214	NM_013249	0.001293
46	ZNF529	NM_020951	0.001293
47	ZNF563	NM_145276	0.001293
48	---	---	0.001293
49	---	---	0.002262
50	---	---	0.002262
51	---	---	0.002262
52	BAZ2A	NM_013449	0.002262
53	CASQ1	NM_001231	0.002262
54	CCDC41	NM_016122	0.002262
55	DACT1	NM_016651	0.002262
56	DDX58	NM_014314	0.002262
57	ERCC1	NM_001983	0.002262
58	FLJ33360	NR_028351	0.002262
59	IFI44	NM_006417	0.002262
60	OAS1	ENST00000202917	0.002262
61	OAS2	ENST00000392583	0.002262
62	PHF8	NM_015107	0.002262
63	RAD9A	NM_004584	0.002262
64	TAF1	NM_004606	0.002262
65	TIAM2	NM_001010927	0.002262
66	USP28	ENST00000003302	0.002262
67	XRCC1	ENST00000458471	0.002262
68	ZFP28	NM_020828	0.002262
69	ZNF256	NM_005773	0.002262
70	ZNF266	NM_006631	0.002262
71	---	---	0.002262
72	---	---	0.002262
73	---	---	0.003878
74	---	---	0.003878
75	---	---	0.003878
76	---	---	0.003878
77	---	---	0.003878
78	---	---	0.003878
79	ATF7	NM_006856	0.003878
80	C17orf65	NR_049729	0.003878
81	C2CD3	NM_015531	0.003878
82	DNAJC9	ENST00000372950	0.003878

83	DYX1C1	NM_130810	0.003878
84	EPHB2	NM_017449	0.003878
85	FAM175A	ENST00000321945	0.003878
86	FHL5	NM_020482	0.003878
87	FMR1	NM_002024	0.003878
88	FOXJ2	ENST00000162391	0.003878
89	GRK4	NM_182982	0.003878
90	IFIT1	NM_001548	0.003878
91	KDM5A	NM_001042603	0.003878
92	KIAA0753	ENST00000361413	0.003878
93	LINC00310	NR_027267	0.003878
94	LOC100132686	BC020894	0.003878
95	LRRTM1	ENST00000433224	0.003878
96	MED31	NM_016060	0.003878
97	MRI1	NM_001031727	0.003878
98	MRPS6	NM_032476	0.003878
99	MTCP1NB	ENST00000369479	0.003878
100	NAF1	NM_138386	0.003878
101	OR52H1	ENST00000322653	0.003878
102	PCDHB13	NM_018933	0.003878
103	PHKA2	ENST00000379942	0.003878
104	PKD1L1	ENST00000462350	0.003878
105	RAB8A	NM_005370	0.003878
106	RIBC1	NM_001267053	0.003878
107	RSAD1	NM_018346	0.003878
108	SLC25A14	NM_003951	0.003878
109	TAB3	NM_152787	0.003878
110	TCTN2	NM_024809	0.003878
111	TINAGL1	NM_022164	0.003878
112	TRDMT1	NM_004412	0.003878
113	TRIM21	NM_003141	0.003878
114	TTC23	NM_001040655	0.003878
115	UBQLNL	NM_145053	0.003878
116	UNC119B	NM_001080533	0.003878
117	ZNF471	NM_020813	0.003878
118	ZNF550	ENST00000325134	0.003878
119	ZNF701	ENST00000301093	0.003878

Table S3. The candidate genes selected from three representative feature sets of 255 features, 50 clinical features and 20 clinical features (corresponding to the Fig. R1 (a), (b) and (d), respectively). *P_100, P_90 and P_80* mean 100%, 90% and 80% of coverage *P*, respectively. The genes from top ranked canonical pathways were in the right column.

255 features (a)			50 clinical features (b)			20 clinical features (d)			Pathway
P_80	P_90	P_100	P_80	P_90	P_100	P_80	P_90	P_100	
AP1M1	AP1M1	AP1M1	AP1M1	AP1M1	C19orf66	AP1M1	AP1M1	IFI44L	IRF9
C19orf66	C19orf66	C19orf66	C19orf66	C19orf66	IFI44L	C19orf66	C19orf66	IFI6	XRCC1
IFI44L	IFI44L	IFI44L	IFI44L	IFI44L	TULP3	IFI44L	IFI44L	BAZ2A	IFIT3
IFIT3	IRF9	IRF9	IRF9	IRF9	APBB3	IRF9	IRF9	ATF7	MRI1
IRF9	TULP3	TULP3	OAS3	TULP3	IFI6	OAS3	TULP3		RAD9A
OAS3	DYNLL2	DYNLL2	TULP3	DYNLL2	MED12	TULP3	DYNLL2		MGLL
TULP3	APBB3	APBB3	DYNLL2	APBB3	BAZ2A	USP18	APBB3		
USP18	IFI6	IFI6	TAS2R19	IFI6	MRPS6	DYNLL2	IFI6		
DYNLL2	MED12	XRCC1	APBB3	MED12		TAS2R19	MED12		
TAS2R19	BAZ2A	ATF7	IFI6	BAZ2A		APBB3	BAZ2A		
APBB3	ERCC1	DNAJC9	MED12	ERCC1		FAM122C	ERCC1		
FAM122C	PHF8	MRPS6	MGLL	PHF8		IFI6	PHF8		
IFI6	XRCC1		BAZ2A	XRCC1		MED12	XRCC1		
MED12	ATF7		ERCC1	ATF7		BAZ2A	ATF7		
MGLL	C2CD3		OAS1	FMR1		ERCC1	FOXJ2		
BAZ2A	DNAJC9		PHF8	FOXJ2		OAS1	MRPS6		
ERCC1	MRPS6		RAD9A	MRPS6		PHF8			
PHF8	RAB8A		XRCC1	TAB3		RAD9A			
RAD9A	TAB3		ATF7	TCTN2		XRCC1			
XRCC1	TCTN2		C17orf65			ATF7			
ATF7			FMR1			DNAJC9			
C17orf65			FOXJ2			FMR1			
C2CD3			KDM5A			FOXJ2			
DNAJC9			MRPS6			KDM5A			
FMR1			RAB8A			MRI1			
FOXJ2			TAB3			MRPS6			
KDM5A			TCTN2			RAB8A			
KIAA0753						TAB3			
MRI1						TCTN2			
MRPS6									
RAB8A									
TAB3									
TCTN2									

Table S4. Predication of MGMT methylation status in previous studies

		Methylated MGMT		Unmethylated MGMT		Sensitivity (1)	Specificity
Brandes, et al[1]	PsP	21	91.3%	11	40.7%	65.6%	88.9%
	TTP	2	8.7%	16	59.3%		
Motegi, et al[2]	PsP	2	40%	1	16.7%	66.7%	62.5%
	TTP	3	60%	5	83.3%		
Kong, et al[3]	PsP	15	37.5%	8	24.2%	65.1%	50%
	TTP	25	62.5%	25	75.8%		
Mieghem, et al[4] (2)	PsP	9	37.5%	4	12.9%	69.2%	64.3%
	TTP	15	62.5%	27	87.1%		
Park, et al[5] (3)	PsP	4	80%	7	35%	36.4%	92.9%
	TTP	1	20%	13	65%		

(1): Sensitivity and specificity were defined for detecting the PsP based on methylated MGMT; (2): MGMT assessed using liberal criteria; (3) MGMT promoter methylation status determined by methylation-specific polymerase chain reaction.

Reference:

1 A.A. Brandes, E. Franceschi, A. Tosoni, V. Blatt, A. Pession, G. Tallini, R. Bertorelle, S. Bartolini, F. Calbucci, A. Andreoli, G. Frezza, M. Leonardi, F. Spagnolli, M. Ermani, "MGMT promoter methylation status can predict the incidence and outcome of pseudoprogression after concomitant radiochemotherapy in newly diagnosed glioblastoma patients," Journal of Clinical Oncology **26**, 2192-2197 (2008).

2 H. Motegi, Y. Kamoshima, S. Terasaka, H. Kobayashi, S. Yamaguchi, M. Tanino, J. Murata, K. Houkin, "IDH1 mutation as a potential novel biomarker for distinguishing pseudoprogression from true progression in patients with glioblastoma treated with temozolomide and radiotherapy," Brain Tumor Pathol **30**, 67-72 (2013).

3 D.S. Kong, S.T. Kim, E.H. Kim, D.H. Lim, W.S. Kim, Y.L. Suh, J.I. Lee, K. Park, J.H. Kim, D.H. Nam, "Diagnostic dilemma of pseudoprogression in the treatment of newly diagnosed glioblastomas: the role of assessing relative cerebral blood flow volume and oxygen-6-methylguanine-DNA methyltransferase promoter methylation status," AJNR Am J Neuroradiol **32**, 382-387 (2011).

4 E. Van Mieghem, A. Wozniak, Y. Geussens, J. Menten, S. De Vleeschouwer, F. Van Calenbergh, R. Sciot, S. Van Gool, O.E. Bechter, P. Demaerel, G. Wilms, P.M. Clement, "Defining pseudoprogression in glioblastoma multiforme," Eur J Neurol **20**, 1335-1341 (2013).

5 C.K. Park, J. Kim, S.Y. Yim, A.R. Lee, J.H. Han, C.Y. Kim, S.H. Park, T.M. Kim, S.H. Lee, S.H. Choi, S.K. Kim, D.G. Kim, H.W. Jung, "Usefulness of MS-MLPA for detection of MGMT promoter methylation in the evaluation of pseudoprogression in glioblastoma patients," Neuro Oncol **13**, 195-202 (2011).

Table S5. Gene expressions of IRF9 and XRCC1 in the samples from TCGA. The sample type, i.e., pseudoprogression (PsP) or true tumor progression (TTP), is determined by the longitudinal MRI from TCIA.

Patient ID	Type (PsP or TTP)	IRF9	XRCC1
TCGA-06-0125	PsP	8.156670711	6.920976104
TCGA-06-0132	TTP	8.478463523	4.926175355
TCGA-06-0138	TTP	7.747738065	5.101606898
TCGA-06-0143	TTP	8.086957245	5.626329777
TCGA-06-0156	TTP	7.578428437	5.655630949
TCGA-06-0166	TTP	7.934104041	5.746312365
TCGA-06-0171	TTP	8.400817171	5.520019494
TCGA-06-0185	PsP	8.894900451	6.044437145
TCGA-06-0188	TTP	8.500423967	5.577020075
TCGA-06-0881	PsP	9.189752485	5.954228976
TCGA-06-1084	TTP	7.645471537	5.196564308
TCGA-14-0783	TTP	8.713694229	5.45244386
TCGA-14-0865	TTP	8.288237374	5.845771704
TCGA-14-1037	PsP	9.226101835	6.326896707
TCGA-14-1402	TTP	8.115959165	5.700478983
TCGA-14-1454	TTP	7.776848448	6.146345705
TCGA-14-1456	PsP	8.399362815	6.416399775
TCGA-14-1459	TTP	7.381387031	5.539365678
TCGA-14-1821	PsP	9.321442885	6.562523335
TCGA-14-1823	TTP	8.29498316	5.399658914
TCGA-14-1829	TTP	7.277918194	5.106995763
